# Use of public health databases for the early identification of Autism spectrum disorder: a scoping review protocol

**DOI:** 10.3389/fdsfr.2026.1816967

**Published:** 2026-06-16

**Authors:** Luiz Jupiter Carneiro de Souza, Luis Phillipe Nagem Lopes, Mariana Del Grossi Moura, Katia Miyuki Sasaki Zeredo, José Fernando Salvador Carrillo, Joaquim Lucas Júnior, Mabel Fernandes Figueiró, Jose Antonio Silvestre Fernandes Neto, Luciane Cruz Lopes, Waldecy Rodrigues

**Affiliations:** 1 Fundação Oswaldo Cruz Foundation, Brasília, Brazil; 2 Institute of Social Medicine, Rio de Janeiro State University, Rio deJaneiro, Brazil; 3 Department of Pharmaceutical Sciences, University of Sorocaba, Sorocaba, Brazil; 4 General Management Coordination, FIOCRUZ Brasilia, Oswaldo Cruz Foundation, Brasilia, Brazil; 5 Escuela Profesional de Medicina Humana, Universidad Privada San Juan Bautista, Bautista, Perú; 6 Heart Hospital of São Paulo, SãoPaulo, Brazil; 7 Federal University of Tocantins, Palmas, Tocantins, Brazil

**Keywords:** autism spectrum disorder, early identification, electronic health records, public health databases, scoping review

## Abstract

**Introduction:**

Diagnosing Autism Spectrum Disorder (ASD) remains a significant challenge for health services worldwide. Delayed diagnosis can lead to negative outcomes for individuals and increased costs for healthcare systems. Utilizing public health databases presents a strategy for identifying clinical characteristics that facilitate the early detection of ASD. This study aims to systematically map the existing evidence on the use of public health databases for the early identification of children and adults with ASD, irrespective of geographic location.

**Methods and Analysis:**

This scoping review will follow the methodological guidelines of the Joanna Briggs Institute. A comprehensive search strategy, validated by a professional librarian, will be conducted across multiple databases, including MEDLINE (via PubMed), EMBASE, Scopus, PsycINFO, Web of Science, and LILACS (via the Virtual Health Library). Gray literature will be reviewed using ProQuest Dissertations and Theses. The review will include only primary studies that utilize public health databases for the early detection of ASD. No restrictions on language or geographic location will be applied. Screening and data extraction will be independently conducted by two trained reviewers, followed by a thematic categorization of studies based on similarities.

**Ethics and Dissemination:**

Ethical approval is not required since this review will exclusively utilize data from published scientific articles. Preliminary findings and final results will be disseminated through presentations at relevant conferences and publications in peer-reviewed journals.

**Registration Number:**

Open Science Framework. DOI: 10.17605/OSF.IO/RMVWE (https://osf.io/rmvwe/).

## Highlights

- The review employs a robust methodology to identify health database sources for early Autism detection.
- Studies will be categorized by country and electronic health record systems to explore disparities in database use for Autism.
- The review excludes biomarker and genetic factor databases, limiting its scope.
- Identifying studies that solely utilize databases for Autism identification may pose methodological challenges.

## Introduction

Autism Spectrum Disorder (ASD) is a neurodevelopmental condition characterized by persistent challenges in social communication and restricted, repetitive patterns of behavior (World Health Organization (WHO), 2023). According to the Centers for Disease Control and Prevention, at least one in every 36 children has been diagnosed with ASD (Maenner et al., 2023). Although these estimates primarily refer to children aged 3–17 years, there is growing evidence that ASD diagnoses are also increasing in adult populations (Howlin and Moss, 2012). Despite this rising prevalence, timely diagnosis remains a major challenge, often delaying access to appropriate mental health and support services and negatively affecting long-term outcomes (Chamak et al., 2011). These barriers are further intensified in low- and middle-income countries, particularly those with universal health systems, where structural and organizational constraints may limit access to specialized diagnostic pathways (Sukiennik et al., 2022).

In this context, the use of routinely collected health data, particularly public health databases, has emerged as a promising approach to support the identification of early signs and indicators of ASD. Such databases, including electronic health records and administrative health systems, enable the large-scale analysis of clinical and epidemiological information that may contribute to earlier recognition of ASD-related patterns (Al-Beltagi, 2023). For example, studies using electronic health record systems have demonstrated the feasibility of identifying individuals with ASD prior to formal diagnosis, with one study reporting that 81% of algorithm-identified cases were later confirmed, probable, or possible ASD diagnoses (Coleman et al., 2015). Similarly, population-based analyses using health system data from large pediatric cohorts have developed predictive algorithms for ASD identification, estimating a prevalence of 1.32% and highlighting the potential of these data sources for epidemiological surveillance and service planning (Brooks et al., 2021).

Beyond early identification, public health databases contribute to multiple dimensions of healthcare systems, including risk factor detection, health service planning, evaluation of healthcare coverage, and prediction of clinical and economic outcomes (Pastorino et al., 2019). However, their use is also accompanied by important challenges, such as ethical concerns related to privacy and autonomy, heterogeneity of data structures, and technical limitations in data integration and infrastructure management (Pastorino et al., 2019). These issues may directly affect the quality, comparability, and scalability of database-driven approaches to ASD research.

Evidence from longitudinal studies further reinforces the relevance of these data sources. For instance, analyses of large integrated health systems have shown distinct trajectories of physical and mental health conditions among autistic youth compared to non-autistic peers, with higher prevalence of obesity, neurological conditions, anxiety, and attention-deficit/hyperactivity disorder (ADHD) across developmental stages (Malow et al., 2023). These findings underscore the importance of systematic monitoring of health conditions in autistic populations and support the potential role of routinely collected data in identifying risk profiles over time.

Recent scoping review synthesized evidence from 1,702 studies and 163 cohorts using health databases in ASD research (Wu et al., 2023). The authors highlighted that these databases are used for multiple purposes, including cohort construction and epidemiological analysis; however, limitations were identified, such as missing data, difficulties in case identification, and inconsistencies in data linkage across systems (Wu et al., 2023). Importantly, the review also showed that the majority of studies originate from high-income countries, indicating a marked geographical imbalance in the production and use of database-driven ASD research (Wu et al., 2023).

In terms of research gaps, it is noteworthy that the scoping review did not focus specifically on the public health databases themselves, but rather on the characteristics of the study populations derived from them (Wu et al., 2023). This limitation highlights the need for studies that explicitly map public health databases used in ASD-related research, with particular attention to their structural characteristics, data generation processes, and potential for supporting early identification of ASD. In addition, the authors emphasize important international disparities in the availability and use of these databases (Wu et al., 2023), suggesting that such differences may reflect broader inequalities in digital health infrastructure and research capacity across countries. These gaps remain insufficiently explored, particularly in relation to how different types of databases are operationalized for early identification purposes in diverse health system contexts.

Building on this gap, although existing literature suggests that the use of public health databases in ASD research is expanding, the evidence remains heavily concentrated in high-income settings. This pattern likely reflects structural inequalities in health information systems, including differences in digital infrastructure, interoperability, data governance, and research investment. However, these disparities have not yet been systematically synthesized according to the types of databases used and their specific applications for early identification of ASD across global contexts.

Thus, the objective of this study is to map the available evidence on the use of public health databases for the early identification of children and adults with ASD, with a specific focus on: (i) the types of databases used (e.g., electronic health records, administrative/claims databases, and registries); (ii) the methodological approaches applied for ASD identification (e.g., screening strategies, predictive models, and algorithm validation); and (iii) the outcomes related to database use, including identification performance, feasibility, and applications for epidemiological surveillance and health service planning, across different geographical contexts.

## Methods and analysis

### Study design

This scoping review will follow the methodological framework recommended by the Joanna Briggs Institute (JBI) (Peters et al., 2024). The review will be reported in accordance with the Preferred Reporting Items for Systematic Reviews and Meta-Analyses Extension for Scoping Reviews (PRISMA-ScR) checklist (Tricco et al., 2018). This protocol was developed following an adapted version of the Preferred Reporting Items for Systematic Review and Meta-Analysis Protocols 2015 checklist (PRISMA-P) for scoping reviews (Supplementary Material 1) (Moher et al., 2015) and was registered in the Open Science Framework (Lopes et al., 2025).

The study will be conducted between May and September 2026. The search strategy will be implemented after protocol registration, and all searches will be performed within a predefined time frame to ensure consistency across databases. The date of the final search will be reported in the full review, and any updates to the search strategy will be documented to ensure transparency and reproducibility.

### Eligibility criteria

#### Participants

We will include studies evaluating individuals diagnosed with or suspected of having ASD, regardless of gender, race, or age group. Studies that do not explicitly use ASD diagnostic codes will also be included if they employ alternative indicators derived from routinely collected health data (e.g., symptom patterns, healthcare utilization, or developmental markers) with the explicit aim of identifying or predicting ASD.

#### Concept

In this scoping review, public health databases are defined as structured systems designed for the systematic collection, storage, integration, and analysis of routinely generated health data, including electronic health records, surveillance systems, and administrative health datasets (Pastorino et al., 2019). These databases are used to support population-level health monitoring and research and include, but are not limited to: (i) electronic health records (EHRs), which capture longitudinal clinical information generated during healthcare delivery; (ii) administrative or claims databases, primarily designed for billing and healthcare utilization tracking; and (iii) disease registries and surveillance systems, which systematically collect data on specific conditions or populations.

These data sources differ substantially in terms of data structure, coding systems, completeness, and validity across settings. Genetic and biomarker-based databases will be excluded from this review because the scope of the study focuses on routinely collected health data embedded within healthcare systems. Although biomarker and genetic data are highly relevant for understanding the etiology and diagnosis of ASD, these sources are typically generated in controlled research or specialized clinical settings and are not routinely available at the population level (Grimberg et al., 2021). Therefore, their inclusion would not align with the objective of mapping scalable, health system–based strategies for the early identification of ASD.

Early identification of ASD is defined as the use of routinely collected health data to detect indicators, patterns, or risk markers associated with ASD prior to, or in support of, formal clinical diagnosis (Al-Beltagi, 2023). This includes approaches based on screening, risk stratification, predictive modelling, and algorithm-based detection using pre-diagnostic information, such as symptoms, healthcare utilization patterns, or developmental indicators. Given the variability in study designs and reporting practices, no fixed age threshold will be applied. Instead, early identification will be operationalized based on the temporal relationship between data use and the diagnostic process, with emphasis on pre-diagnostic or diagnostic-support phases.

#### Context

No restrictions will be applied regarding geographical location or healthcare setting (primary, secondary, or tertiary care).

#### Inclusion and exclusion criteria

We will include studies that explicitly use public health databases to support the early identification of ASD, defined as approaches aimed at detecting, predicting, or flagging ASD-related patterns prior to, or in support of, formal diagnosis. Eligible study designs may include predictive modelling studies, validation studies of identification algorithms, screening approaches, and database-driven detection strategies.

Studies will be excluded if they: (i) focus exclusively on epidemiological descriptions (e.g., prevalence or incidence) without an identification component; (ii) rely solely on genetic or biological data; or (iii) do not involve the use of routinely collected health data.

### Search strategy

The selection of databases was guided by their relevance to biomedical, psychological, and multidisciplinary health research, ensuring comprehensive and systematic coverage of both clinical and public health literature. The following databases will be searched: MEDLINE (via PubMed), EMBASE, Scopus, PsycINFO, Web of Science, and LILACS (via the Virtual Health Library portal). These databases were selected to capture both global and Latin American evidence and to reflect the international and regional scope of ASD research.

In addition, grey literature sources, including ProQuest Dissertations and Theses, will be searched to reduce publication bias and identify methodological approaches and innovations not yet available in peer-reviewed journals. The search strategy will combine controlled vocabulary and free-text terms and was developed and validated in collaboration with an experienced information specialist librarian (McGowan et al., 2016; Rethlefsen et al., 2021).

The concept of early identification was operationalized in the search strategy using a combination of terms related to screening, early detection, risk identification, and predictive modelling, together with ASD-related descriptors. This approach aims to ensure sensitivity in identifying studies that use routinely collected health data to detect patterns associated with ASD prior to formal diagnosis. The full search strategy, including all search terms and combinations, is provided in Supplementary Material 2.

To enhance comprehensiveness, additional sources will include the reference lists of all included studies, consultation with experts in the field, and citations from previously published scoping and systematic reviews.

### Study selection and data extraction

Studies will be screened by title and abstract independently by two reviewers using Rayyan, a web-based application designed to facilitate study screening and selection in systematic and scoping reviews, following the automatic removal of duplicates (Ouzzani et al., 2016). Full-text screening will follow a similar process but will be conducted in Microsoft Excel, and reasons for exclusion will be systematically recorded.

Data extraction will be conducted using a standardized and pilot-tested form. In addition to Population, Concept, and Context variables, we will extract detailed information on: (i) type and characteristics of the database (e.g., electronic health records, administrative databases, surveillance systems); (ii) data sources and structure; (iii) methods used for ASD identification (e.g., algorithms, diagnostic codes, predictive models); (iv) stage of identification (e.g., screening, pre-diagnostic identification, confirmed diagnosis); (v) outcomes related to database use (e.g., accuracy, feasibility, epidemiological estimates); and (vi) geographical and healthcare system context.

Data extraction will also include a detailed classification of the type of database used in each study, including electronic health records, administrative/claims databases, registries, and surveillance systems. The complete list of extracted variables is provided in Supplementary Material 3.

Before each review stage, the reviewers will conduct a pilot calibration exercise. Reviewers will be considered calibrated when at least 80% agreement is achieved during screening, full-text selection, and data extraction.

### Data categorization and analysis

Data will be analyzed using a structured narrative synthesis complemented by descriptive quantitative summaries. First, databases will be categorized according to type (e.g., electronic health records, administrative databases, surveillance systems), geographic origin, and healthcare system level. Second, we will map the intended use of each database (e.g., early identification, epidemiological surveillance, service planning, predictive modelling). Third, studies will be stratified according to World Bank country income classifications to explore potential global disparities (World Bank Country and Lending Groups, 2024). Finally, findings will be synthesized into thematic categories reflecting methodological approaches, data structures, and applications for ASD identification. Descriptive statistics will be used to summarize the findings, and analyses will be conducted in Microsoft Excel.

To address heterogeneity in study objectives, included studies will be categorized according to their primary analytical purpose, such as: (i) early identification or screening; (ii) predictive modelling; (iii) validation of identification algorithms; and (iv) surveillance-related detection strategies. This categorization will support structured comparisons across studies with different methodological aims.

## Discussion

The results of this scoping review will allow us to compile a set of public health databases used for the early identification of ASD, as well as to examine regional disparities worldwide in the use of this resource (Mandell et al., 2007). This is particularly important given that the diagnosis of ASD is currently based exclusively on clinical evaluation, requiring the expertise and training of medical professionals (Smith-Young et al., 2024).

The use of public health databases to identify the clinical characteristics of user populations helps health systems mitigate barriers to diagnostic access (Smith-Young et al., 2024). Additionally, it supports surveillance and community screening efforts, which are essential for improving health outcomes in populations with ASD (Pastorino et al., 2019).

Beyond mapping the use of public health databases in ASD research, this review may also contribute to a broader understanding of global inequalities in digital health infrastructure and research capacity. The concentration of studies in high-income countries may reflect disparities in the availability of interoperable health information systems, standardized data governance frameworks, and investments in digital health technologies. In addition, the heterogeneity identified across database types, coding practices, and analytical approaches may indicate important methodological fragmentation in ASD identification research. These findings may help inform future efforts toward greater standardization, interoperability, and equitable development of database-driven strategies for ASD identification across diverse healthcare system contexts.

Limitations may arise in this scoping review, particularly regarding the heterogeneity in how studies report on public health databases and the variability in the populations included in the studies (Wu et al., 2023). Furthermore, the relatively recent adoption of early identification practices for ASD (Al-Beltagi, 2023) presents challenges in refining the search strategy to identify studies specifically utilizing public health databases for this purpose. These challenges will be mitigated by employing robust methodological procedures, developing a comprehensive data extraction form, and relying on a team with expertise in the proposed method, who will be thoroughly trained in applying the eligibility criteria.
