# Supplementary material for: Use of public health databases for the early identification of Autism spectrum disorder: a scoping review protocol
Source: Front Drug Saf Regul. 2026 Jun 16;6:1816967. doi: 10.3389/fdsfr.2026.1816967 (PMC13317197; doi:10.3389/fdsfr.2026.1816967)
Supplement: Supplementary file 2 [file Supplementaryfile3.docx]

**Supplemental material 3.** Data Extraction

**Part 1. Study Characteristics:**

1. **Study Design** (e.g., observational, cohort, cross-sectional, case-control, etc.):
2. **Geographical Location** (Country/Region) and World Bank classification^[[1]](#footnote-1)^
3. **Target Population:**
   - Children
   - Adults
   - Both
4. **Age Range:**
   - Minimum age
   - Maximum age
5. **Sample Size:**
   - Number of participants
   - Number of individuals diagnosed with ASD (if reported)

**Part 2. Public Health Database Characteristics:**

1. **Database(s) Used:**
   - Name of database(s) (e.g., electronic health records, public health surveillance systems, national registries, etc.)
2. **Database Access Type:**
   - Open access
   - Restricted access
3. **Purpose of Database Use:**
   - Early identification of ASD
   - Surveillance
   - Screening
   - Diagnosis
   - Other (please specify)
4. **Database Characteristics:**
   - Type of data included (e.g., health records, demographic data, clinical records, survey data)
   - Temporal coverage (start and end dates)
   - Geographical scope (local, regional, national, international)

**Part 3. Methods and Analysis:**

1. **Diagnostic Criteria for ASD:**
   - Clinical diagnosis (e.g., DSM-5, ICD-10)
   - Standardized screening tools (e.g., ADOS, M-CHAT)
   - Other (please specify)
2. **Methodology for Early Identification:**
   - Description of algorithms, screening tools, or criteria used for ASD identification
   - Technologies used for early identification: e.g. algorithm, predictive model, among others
   - common health conditions of the patients surveyed: e.g. previous diagnoses of mental disorders, intellectual disability, among others
3. **Integration of Health Database for Early Identification:**
   - Integration with clinical or diagnostic services
   - Use in public health surveillance or epidemiological studies
4. **Techniques Used** (if applicable):
   - Descriptive statistics
   - Regression models
   - Machine learning techniques

**Part 4. Key Findings and Outcomes:**

1. **Main Findings:**
   - Key results related to the use of public health databases in identifying ASD early
2. **Effectiveness of Early Identification:**
   - Success rate, sensitivity, and specificity (if reported)
   - Barriers to effective use of databases for early identification
3. **Disparities in ASD Diagnosis:**
   - Identified disparities based on geography, age, or other sociodemographic factors
4. **Limitations Identified by the Study:**
   - Limitations in database access, data quality, or methodology

1. For the current 2025 fiscal year, low-income economies are defined as those with a Gross national income per capita, calculated using the World Bank Atlas method, of $1,145 or less in 2023; lower middle-income economies are those with a Gross national income per capita between $1,146 and $4,515; upper middle-income economies are those with a Gross national income per capita between $4,516 and $14,005; high-income economies are those with more than a Gross national income per capita of $14,005. [↑](#footnote-ref-1)
