# Supplementary material for: Use of public health databases for the early identification of Autism spectrum disorder: a scoping review protocol
Source: Front Drug Saf Regul. 2026 Jun 16;6:1816967. doi: 10.3389/fdsfr.2026.1816967 (PMC13317197; doi:10.3389/fdsfr.2026.1816967)
Supplement: Supplementary file 3 [file Supplementaryfile2.docx]

**Supplemental material 2.** Search Strategy

**MEDLINE (Via Pubmed)**

| **ID** | **Search Strategy** | **Results** |
| --- | --- | --- |
| 01 | “Autism Spectrum Disorder”[MH] OR “Autistic Spectrum Disorder” OR “Autistic Spectrum Disorders” OR “Disorder, Autistic Spectrum” OR “Autism Spectrum Disorders” OR “Autistic Disorder”[MH] OR “Disorder, Autistic” OR “Disorders, Autistic” OR Autism OR “Autism, Early Infantile” OR “Early Infantile Autism” OR “Infantile Autism, Early” OR “Autism, Infantile” OR “Infantile Autism” OR “Kanner's Syndrome” OR “Kanners Syndrome” OR “Kanner Syndrome” | 81,553 |
| 02 | “Electronic Health Records”[MH] OR “Health Record, Electronic” OR “Health Records, Electronic” OR “Electronic Medical Record” OR “Medical Record, Electronic” OR “Medical Records, Electronic” OR “Electronic Health Record” OR “Electronic Medical Records” OR “Medical Records, Computerized” OR “Computerized Medical Record” OR “Computerized Medical Records” OR “Medical Record, Computerized” OR “Electronic Health Record Data” OR “statistics and numerical data” OR “Sentinel Surveillance”[MH] OR “Surveillance, Sentinel” OR “Biosurveillance Systems” OR “Biosurveillance System” OR “Syndromic Surveillance” OR “Surveillance, Syndromic” OR “Sentinel Health Event” OR “health databases” | 1,164,823 |
| 03 | 01 AND 02 | 3,005 |

**EMBASE**

| **ID** | **Search Strategy** | **Results** |
| --- | --- | --- |
| 01 | ('autism'/exp OR 'autistic spectrum disorder' OR 'autistic spectrum disorders' OR 'disorder, autistic spectrum' OR 'autism spectrum disorders' OR 'autism'/exp OR 'disorder, autistic' OR 'disorders, autistic' OR 'autism' OR 'autism, early infantile' OR 'early infantile autism' OR 'infantile autism, early' OR 'autism, infantile' OR 'infantile autism' OR 'kanner`s syndrome' OR 'kanners syndrome' OR 'kanner syndrome') | 124,087 |
| 02 | ('electronic health record'/exp OR 'health record, electronic' OR 'health records, electronic' OR 'electronic medical record' OR 'medical record, electronic' OR 'medical records, electronic' OR 'electronic health record' OR 'electronic medical records' OR 'medical records, computerized' OR 'computerized medical record' OR 'computerized medical records' OR 'medical record, computerized' OR 'electronic health record data' OR 'statistics and numerical data' OR 'sentinel surveillance'/exp OR 'surveillance, sentinel' OR 'biosurveillance systems' OR 'biosurveillance system' OR 'syndromic surveillance' OR 'surveillance, syndromic' OR 'sentinel health event' OR 'health databases') | 259,804 |
| 03 | #01 AND #02 AND [embase]/lim NOT ([embase]/lim AND [medline]/lim) | 241 |

**Scopus**

| **ID** | **Search Strategy** | **Results** |
| --- | --- | --- |
| 01 | TITLE-ABS-KEY ( "Autism Spectrum Disorder" OR "Autistic Spectrum Disorder" OR "Autistic Spectrum Disorders" OR "Disorder, Autistic Spectrum" OR "Autism Spectrum Disorders" OR "Autistic Disorder" OR "Disorder, Autistic" OR "Disorders, Autistic" OR autism OR "Autism, Early Infantile" OR "Early Infantile Autism" OR "Infantile Autism, Early" OR "Autism, Infantile" OR "Infantile Autism" OR "Kanner's Syndrome" OR "Kanners Syndrome" OR "Kanner Syndrome" ) | 127,647 |
| 02 | TITLE-ABS-KEY ( "Electronic Health Records" OR "Health Record, Electronic" OR "Health Records, Electronic" OR "Electronic Medical Record" OR "Medical Record, Electronic" OR "Medical Records, Electronic" OR "Electronic Health Record" OR "Electronic Medical Records" OR "Medical Records, Computerized" OR "Computerized Medical Record" OR "Computerized Medical Records" OR "Medical Record, Computerized" OR "Electronic Health Record Data" OR "statistics and numerical data" OR "Sentinel Surveillance" OR "Surveillance, Sentinel" OR "Biosurveillance Systems" OR "Biosurveillance System" OR "Syndromic Surveillance" OR "Surveillance, Syndromic" OR "Sentinel Health Event" OR "health databases" ) | 383,496 |
| 03 | #01 AND #02 | 1,146 |

**LILACS (Via the Portal da Biblioteca Virtual de Saúde)**

| **ID** | **Search Strategy** | **Results** |
| --- | --- | --- |
| 01 | (Autism Spectrum Disorder) OR (Autistic Spectrum Disorder) OR (Autistic Spectrum Disorders) OR (Disorder, Autistic Spectrum) OR (Autism Spectrum Disorders) OR (Autistic Disorder) OR (Disorder, Autistic) OR (Disorders, Autistic) OR Autism OR (Autism, Early Infantile) OR (Early Infantile Autism) OR (Infantile Autism, Early) OR (Autism, Infantile) OR (Infantile Autism) OR (Kanner's Syndrome) OR (Kanners Syndrome) OR (Kanner Syndrome) | 4,483 |
| 02 | (Electronic Health Records) OR (Health Record, Electronic) OR (Health Records, Electronic) OR (Electronic Medical Record) OR (Medical Record, Electronic) OR (Medical Records, Electronic) OR (Electronic Health Record) OR (Electronic Medical Records) OR (Medical Records, Computerized) OR (Computerized Medical Record) OR (Computerized Medical Records) OR (Medical Record, Computerized) OR (Electronic Health Record Data) OR (statistics and numerical data) OR (Sentinel Surveillance) OR (Surveillance, Sentinel) OR (Biosurveillance Systems) OR (Biosurveillance System) OR (Syndromic Surveillance) OR (Surveillance, Syndromic) OR (Sentinel Health Event) OR (health databases) | 110,875 |
| 03 | #01 AND #02 | 175 |

**PsycInfo**

| **ID** | **Search Strategy** | **Results** |
| --- | --- | --- |
|  | **Any Field**: "Autism Spectrum Disorder" *OR* **Any Field**: "Autistic Spectrum Disorder" *OR* **Any Field**: "Autistic Spectrum Disorders" *OR* **Any Field**: "Disorder, Autistic Spectrum" *OR* **Any Field**: "Autism Spectrum Disorders" *OR* **Any Field**: "Autistic Disorder" *OR* **Any Field**: "Disorder, Autistic" *OR* **Any Field**: "Disorders, Autistic" *OR* **Any Field**: autism *OR* **Any Field**: "Autism, Early Infantile" *OR* **Any Field**: "Early Infantile Autism" *OR* **Any Field**: "Infantile Autism, Early" *OR* **Any Field**: "Autism, Infantile" *OR* **Any Field**: "Infantile Autism" *OR* **Any Field**: "Kanner's Syndrome" *OR* **Any Field**: "Kanners Syndrome" *OR* **Any Field**: "Kanner Syndrome" | 103,094 |
|  | **Any Field**: "Electronic Health Records" *OR* **Any Field**: "Health Record, Electronic" *OR* **Any Field**: "Health Records, Electronic" *OR* **Any Field**: "Electronic Medical Record" *OR* **Any Field**: "Medical Record, Electronic" *OR* **Any Field**: "Medical Records, Electronic" *OR* **Any Field**: "Electronic Health Record" *OR* **Any Field**: "Electronic Medical Records" *OR* **Any Field**: "Medical Records, Computerized" *OR* **Any Field**: "Computerized Medical Record" *OR* **Any Field**: "Computerized Medical Records" *OR* **Any Field**: "Medical Record, Computerized" *OR* **Any Field**: "Electronic Health Record Data" *OR* **Any Field**: "statistics and numerical data" *OR* **Any Field**: "Sentinel Surveillance" *OR* **Any Field**: "Surveillance, Sentinel" *OR* **Any Field**: "Biosurveillance Systems" *OR* **Any Field**: "Biosurveillance System" *OR* **Any Field**: "Syndromic Surveillance" *OR* **Any Field**: "Surveillance, Syndromic" *OR* **Any Field**: "Sentinel Health Event" *OR* **Any Field**: "health databases" | 51 |
| 03 | #01 AND #02 | 178 |

**Web of Science**

| **ID** | **Search Strategy** | **Results** |
| --- | --- | --- |
| 01 | ALL=(("Autism Spectrum Disorder" OR "Autistic Spectrum Disorder" OR "Autistic Spectrum Disorders" OR "Disorder, Autistic Spectrum" OR "Autism Spectrum Disorders" OR "Autistic Disorder" OR "Disorder, Autistic" OR "Disorders, Autistic" OR autism OR "Autism, Early Infantile" OR "Early Infantile Autism" OR "Infantile Autism, Early" OR "Autism, Infantile" OR "Infantile Autism" OR "Kanner's Syndrome" OR "Kanners Syndrome" OR "Kanner Syndrome")) | 117,928 |
| 02 | ALL=("Electronic Health Records" OR "Health Record, Electronic" OR "Health Records, Electronic" OR "Electronic Medical Record" OR "Medical Record, Electronic" OR "Medical Records, Electronic" OR "Electronic Health Record" OR "Electronic Medical Records" OR "Medical Records, Computerized" OR "Computerized Medical Record" OR "Computerized Medical Records" OR "Medical Record, Computerized" OR "Electronic Health Record Data" OR "statistics and numerical data" OR "Sentinel Surveillance" OR "Surveillance, Sentinel" OR "Biosurveillance Systems" OR "Biosurveillance System" OR "Syndromic Surveillance" OR "Surveillance, Syndromic" OR "Sentinel Health Event" OR "health databases") | 76,302 |
| 03 | #01 AND #02 | 321 |

**ProQuest**

| **ID** | **Search Strategy** | **Results** |
| --- | --- | --- |
| 01 | ("Autism Spectrum disorder" OR Autism) | 4,695 |
| 02 | ("Electronic Health Records" OR "Electronic Medical Records" OR "health databases") | 1,375 |
| 03 | #01 AND #02 AND Filter: Theses and Dissertations | 102 |
